# Decoding Lodging Resistance in Elite Chinese Conventional Rice Varieties: A Phenotypic and Biomechanical Perspective

**DOI:** 10.3390/plants14182878

**Published:** 2025-09-16

**Authors:** Yufei Li, Lu Zhou, Fan Zhu, Yinmei Tang, Qun Ni, Jing Ren, Biyu Huang, Zhenqian Zhang, Yue Wang, Yulin Peng

**Affiliations:** 1 Yuelu Mountain Laboratory, Changsha 410128, China; lyf5998@stu.hunau.edu.cn; 2 Department of Agronomy, College of Agronomy, Hunan Agricultural University, Changsha 410128, China; zhoulu@stu.hunau.edu.cn (L.Z.); 562560172@stu.hunau.edu.cn (F.Z.); tym7044@stu.hunau.edu.cn (Y.T.); niqun@stu.hunau.edu.cn (Q.N.); renjing111@stu.hunau.edu.cn (J.R.); 200418a@stu.hunau.edu.cn (B.H.); zhangzhenqian@hunau.edu.cn (Z.Z.); 3 Hunan Hybrid Rice Research Center, No. 736, Yuanda Road, Furong District, Changsha 410125, China

**Keywords:** high-quality, conventional rice, lodging characteristics, lodging

## Abstract

The lodging resistance of rice is a prerequisite for ensuring yield and rice quality. An in-depth analysis of key traits affecting rice lodging resistance is crucial for guiding the cultivation of excellent rice varieties and field production. Given consumer demand for high-quality rice and frequent extreme weather conditions, this study focused on six high-quality conventional rice varieties and compared the main stem internode physical traits, stem and sheath plumpness traits, main stem mechanical properties, yield-related traits, and panicle characteristics of the plants based on field phenotype measurements. Among them, three varieties showed lodging resistance in the field, while the other three varieties all experienced varying degrees of lodging susceptibility. The results showed that lodging-resistant varieties exhibited a more reasonable internode structure, lower plant height, gravity center height, and relative gravity center height, as well as shorter and thicker second internodes (N2). Additionally, they had higher sheath phimosis degree, greater bending stress, internode-breaking moment, and plant-breaking moment, along with a lower lodging index compared to lodging-susceptible varieties. Specifically, lodging-resistant varieties had 0.83–9.61% lower plant height, 4.11–16.10% lower gravity center height, and 0.09–12.68% lower relative gravity center height than those of lodging-susceptible varieties. Their N2 internode length was 8.96–44.69% shorter, while stem and sheath weight ratios were 16.37–268.58% and 8.27–165.01% higher than those of lodging-susceptible varieties, respectively. At the same time, lodging-resistant varieties exhibit the ability to stabilize yield while reducing their own risk of lodging by increasing effective panicles and reducing single panicle weight. In addition, NX42, LD3, and SY17 were ultimately evaluated as low-risk lodging varieties in this study. This study aims to address the lodging problem of high-quality conventional rice and analyze the key mechanisms underlying its lodging resistance. The research provides important theoretical support for genetic improvement of high-quality conventional rice.

## 1. Introduction

Rice is a staple food and primary energy source for nearly half of the world’s population. It has a significant impact on nutrition and health [1]. With the socioeconomic development accompanied by continuous improvement in living standards, people pay more attention to and pursue the quality and safety of food. Consumers’ demand for rice has also shifted from quantity to high quality [2]. But for a long time, high yield has been regarded as the main goal of rice breeding to meet the rapid growth of the world’s population [3]. At the same time, there is a contradiction between achieving high yield and high quality of rice. Most high-yield hybrid rice varieties exhibit a decline in rice quality. After cooking, the rice has high hardness and poor viscosity [4]. But with further innovation in breeding technology, breeders are accelerating the selection of high-quality rice varieties. The overall objective is to maintain excellent rice quality on the basis of yield and resistance properties to meet the needs of rice production [5].

Currently, there is a significant increase in the frequency, intensity, and duration of strong convective weather, including extreme heat and extreme rainfall, globally. This has become the most serious challenge to food production. Extreme precipitation indicators were significantly and negatively correlated with rice yields in Jiangsu and Hainan Provinces, China [6]. Related studies also show that under different climate changes, extreme heat events and extreme precipitation events in rice-producing areas are on the rise in the future [7]. Rainfall is often accompanied by strong winds. Strong winds exceeding the carrying capacity of rice stems will directly lead to rice lodging. If the soil moisture is too high, the roots cannot firmly root. Combined with strong winds, the risk of rice lodging will further increase.

Plant structure is one of the important agronomic traits that determines the formation of rice grain yield and quality. It is mainly influenced by factors such as tillering, stem characteristics, and panicle morphology. Effective tillering ensures an effective number of panicles, which is crucial for achieving high crop yields [8]. Dwarf phenotype is beneficial for rice to resist lodging. But if the plant is too short, it will lead to insufficient growth, ultimately affecting the yield potential of rice. So, under low lodging risk, it is necessary to increase plant height to improve yield [9]. Further analysis was conducted on non-structural carbohydrates (NSCs) in rice stem sheaths. The results revealed that these stored NSCs participate in grain filling. Moreover, they significantly influence both the initiation and enrichment of grain filling. Consequently, NSCs further affect rice quality. A significant positive correlation was observed between the brown rice rate and the accumulation of soluble sugars and starch in the panicle. The chalkiness degree was significantly or highly significantly correlated with the soluble sugar accumulation in stems and leaves [10]. And lignocellulose, as a structural carbohydrate, is the key to crop lodging resistance. Plant stems not only provide transportation of water and nutrients but also provide rigidity and strength for plants to resist external environments [11]. Among them, lignin content is significantly correlated with the mechanical strength of cell walls and the lodging resistance of stems. Compared to cellulose, the lignin content in stems has a greater impact on stem strength. Its content can be used as an indicator to evaluate crop lodging resistance [12]. In addition, a reasonable panicle shape can reduce the risk of crop lodging. The increase in grain weight per panicle and plant height of heavy panicle rice varieties may lead to an increase in bending moment, which may increase the risk of lodging. But if the bending resistance of rice is significantly improved, the overall stem lodging resistance will not be significantly reduced [13]. Rice populations with smaller panicle types and more effective panicles can achieve dual improvements in high yield and lodging resistance [14]. In northern japonica rice, the lodging index of upright and semi upright panicle type rice varieties is smaller than that of curved panicle type rice varieties, indicating better lodging resistance [15]. Therefore, the lodging resistance of rice is a complex comprehensive trait.

The conclusions of different studies on key traits affecting rice lodging vary. The mechanical judgment method and model evaluation method are the main international evaluation methods for the lodging resistance of rice stems [16,17,18]. Among them, lodging index and internode bending resistance are the main indicators for evaluating the lodging resistance of rice using mechanical judgment methods [19]. But some studies suggest that compared to the lodging index evaluation, assessing rice’s lodging resistance based on the physical strength of the stem may be more accurate [20,21]. Other researchers suggest that the internode bending resistance is not perfect in demonstrating the lodging resistance of rice stems. The actual lodging resistance of rice should be reflected in the overall thrust resistance of the crop itself [22,23,24,25]. With the development and application of crop models, crop growth patterns have shifted towards quantitative analysis, which has led to more attention being paid to the study of lodging models [26,27,28,29]. But model evaluation is usually based on some simplified assumptions. These assumptions may deviate from the actual situation, leading to uncertainty in the evaluation results. Meanwhile, model construction requires a large amount of data support. If the data is not comprehensive or accurate enough, it will affect the reliability of the evaluation results. In addition, the selection of parameters in model evaluation has a significant impact on the results. But sometimes the selection of these parameters may be subjective or based on experience. The credibility of the evaluation results is low. This results in the stem mechanics evaluation method still being the most widely used and effective evaluation method in practical applications. However, there is little research on the influence of rice panicle characteristic on rice lodging, as well as the correlation between rice panicle characteristic indicators and stem characteristic indicators.

Therefore, this study focuses on the lodging characteristics of six high-quality conventional rice varieties in the context of consumer demand for high-quality rice and frequent extreme weather events. Three varieties showed no lodging in the field, while three varieties experienced varying degrees of lodging. Based on field performance, we compared a range of main stem attributes, including the following: internode physical traits, stem and sheath plumpness, mechanical properties, yield-related traits, and panicle characteristics. The purpose is to (1) clarify the lodging resistance mechanism of high-quality conventional rice varieties; (2) search for key indicators to evaluate the lodging resistance of high-quality conventional rice, and (3) construct the relationship between panicle characteristic indicators and stem lodging indicators. The research ultimately provides theoretical reference for the breeding and production application of high-quality conventional rice.

## 2. Results

### 2.1. Comparison of Main Stem Internode Physical Traits of Different High-Quality Conventional Rice

#### 2.1.1. Plant Height and Internode Length

**Figure 1:**
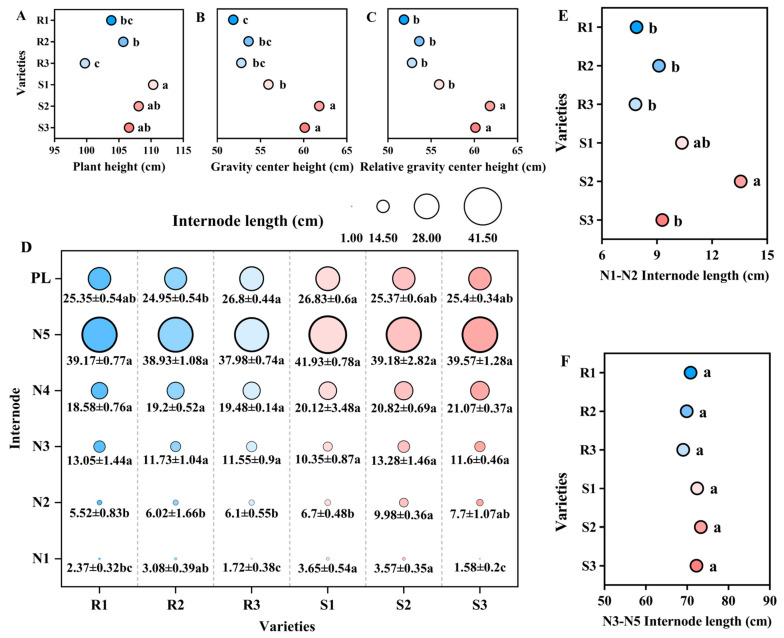
Comparison of main stem height and internode length of different high-quality conventional rice varieties. (**A**) Plant height (cm); (**B**) gravity center height (cm); (**C**) relative gravity center height (%); (**D**) Internode length (cm); (**E**) N1–N2 internode length (cm); (**F**) N3–N5 internode length (cm). R1–R3 represent lodging-resistant varieties, S1–S3 represent lodging-susceptible varieties. PL represents panicle length, N1–N5 represent the 1st to 5th internodes from the base upwards, respectively. Different lowercase letters represent statistically significant differences between varieties for the same indicator.

Both plant height and gravity center height of the lodging-resistant varieties were smaller than those of the lodging-susceptible varieties. Among them, the plant height of SY17 (R3) was significantly lower than JXSM (S1) by 9.61%, and gravity center height of NX42 (R1) was significantly lower than HGNZ (S2) by 16.10%. The difference in relative gravity center height between NX42 (R1) and HGNZ (S2) is also most significant. The former is significantly lower than the latter by 12.68% (Figure 1A–C). Further analysis of the internode length and panicle length of the main stem revealed that there was no significant difference in internode length between lodging-resistant and lodging-susceptible varieties for N3, N4, and N5 of the main stem. The internode length of the N2 on the main stem of lodging-resistant varieties is smaller than that of lodging-susceptible varieties. Among them, NX42 (R1), LD3 (R2), and SY17 (R3) were significantly smaller than HGNZ (S2), reaching 44.69%, 39.68%, and 38.88%, respectively (Figure 1D). Further stacking of the internodes revealed that the lengths of N1-N2 and N3-N5 internodes were shorter in lodging-resistant varieties than in lodging-susceptible varieties. And there is a significant difference in the accumulation between N1-N2 internodes. The differences still show that NX42 (R1), LD3 (R2), and SY17 (R3) are significantly smaller than HGNZ (S2), reaching 32.84%, 41.82%, and 42.31%, respectively. Therefore, lodging-resistant varieties may have more reasonable internode length and configuration of the main stem. Among them, the influence of N2 and N1-N2 is the most significant (Figure 1E,F).

#### 2.1.2. Internode Physical Traits

**Figure 2:**
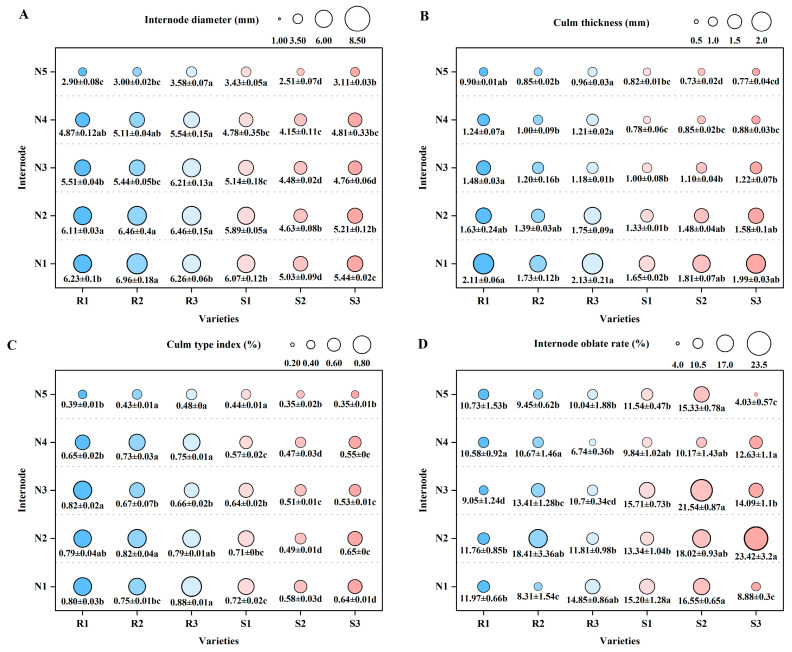
Comparison of main stem internode physical traits of different high-quality conventional rice varieties. (**A**) Internode diameter (mm); (**B**) culm thickness (mm); (**C**) culm type index (%); (**D**) internode oblate rate (%). R1–R3 represent lodging-resistant varieties, S1–S3 represent lodging-susceptible varieties. N1–N5 represent the 1st to 5th internodes from the base upwards, respectively. Different lowercase letters represent statistically significant differences between varieties for the same indicator.

The internode diameter between N1 to N4 is greater in lodging-resistant varieties than in lodging-susceptible varieties. Among them, the difference in internode diameter between the N1 and N2 internodes of the main stem was most significant between LD3 (R2) and HGNZ (S2). The former is significantly higher than the latter, reaching 38.37% and 39.52%, respectively. The difference in internode diameter between the N3 and N4 internodes of the main stem is most significant between SY17 (R3) and HGNZ (S2). The former is significantly higher than the latter, reaching 38.62% and 33.49%, respectively. There is no significant difference in culm thickness between the N1 to N3 internodes of the main stem between the two varieties. But in both N4 and N5 internodes, the culm thickness of lodging-resistant varieties is greater than that of lodging-susceptible varieties (Figure 2A,B). Based on the differences in internode length and culm thickness, further compare the culm type index and internode oblate rate of each internode of the main stem of different varieties. The results showed that the culm type index of the N1-to-N4 internodes of the main stem was higher in lodging-resistant varieties than in lodging-susceptible varieties. The N1 internodes of NX42 (R1) and SY17 (R3) were significantly higher than those of lodging-susceptible varieties, reaching 11.11–37.93% and 22.22–51.72%, respectively. Among the N2 internodes, LD3 (R2) was significantly higher than the lodging-susceptible variety, reaching 15.49–67.35%. Among N3 internodes, NX42 (R1) was significantly higher than the lodging-susceptible variety, reaching 28.13–60.78%. The lodging-resistant varieties between N3 internodes were significantly larger than the lodging-susceptible varieties. The internode oblate rate was only observed in the N3 internodes of the main stem, and the lodging-resistant varieties were all lower than the lodging-susceptible varieties (Figure 2C,D). This indicates that the differences in physical traits between lodging-resistant varieties and lodging-susceptible varieties are mainly reflected in internode diameter and culm type index.

### 2.2. Comparison of Stem and Sheath Plumpness Traits of Different High-Quality Conventional Rice

**Figure 3:**
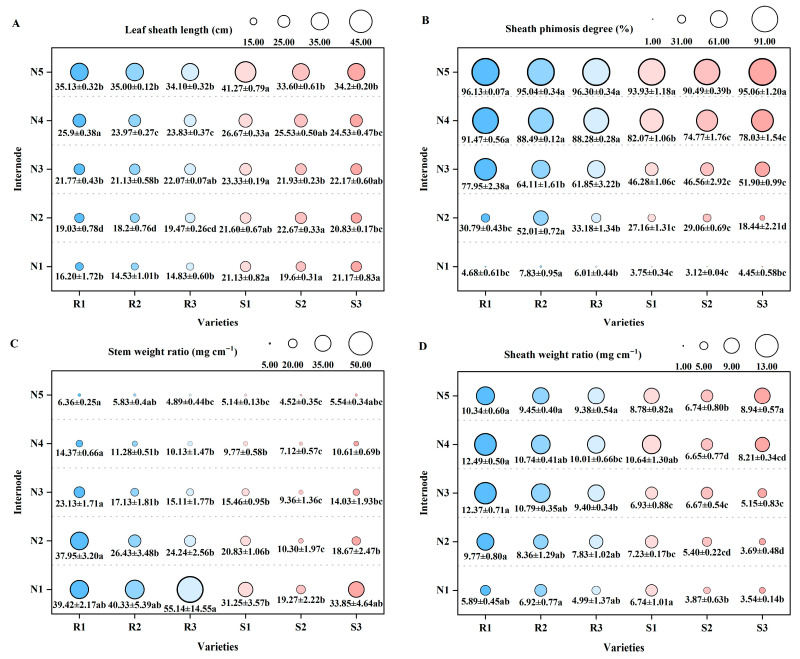
Comparison of stem and sheath plumpness traits of different high-quality conventional rice varieties. (**A**) Leaf sheath length (cm); (**B**) sheath phimosis degree (%); (**C**) stem weight ratio (mg cm^−1^); (**D**) sheath weight ratio (mg cm^−1^). R1–R3 represent lodging-resistant varieties, S1–S3 represent lodging-susceptible varieties. N1–N5 represent the 1st to 5th internodes from the base upwards, respectively. Different lowercase letters represent statistically significant differences between varieties for the same indicator.

The leaf sheath length and sheath phimosis degree of the main stem increase with the increase in node position from N1 to N5 internodes. Meanwhile, the leaf sheath length between the N1 and N2 internodes of lodging-resistant varieties is smaller than that of lodging-susceptible varieties, but the sheath phimosis degree is greater than lodging-susceptible varieties. The sheath phimosis degree reflects the degree to which the leaf sheath wraps around the stem. From this, it can be seen that the leaf sheaths of lodging-resistant varieties have a greater impact on plant resistance to lodging than lodging-susceptible varieties (Figure 3A,B). In addition, between the N1 and N4 internodes of the main stem, the stem weight ratio of each variety decreases with increasing node position, while the sheath weight ratio increases with increasing node position. Meanwhile, the stem weight ratio of internodes N1 to N4 on the main stem of lodging-resistant varieties is greater than lodging-susceptible varieties. And the sheath weight ratio between N2-to-N5 internodes is greater in lodging-resistant varieties than in lodging-susceptible varieties. Among them, there are significant differences in stem and sheath plumpness traits at N2 internodes. Specifically, the stem weight ratio and sheath weight ratio of NX42 (R1) was significantly higher than JXSM (S1), HGNZ (S2), and HGYZ (S3), reaching 82.19% and 35.13%, 268.45% and 80.93%, and 103.27% and 164.77%, respectively (Figure 3C,D). In summary, compared to lodging-susceptible varieties, lodging-resistant varieties have a higher sheath phimosis degree, and higher stem weight ratio and sheath weight ratio. Especially the N2 internode, which has good internode fullness.

### 2.3. Comparison of Main Stem Mechanical Properties and Lodging Resistance of Different High-Quality Conventional Rice

#### 2.3.1. Main Stem Mechanical Properties

**Figure 4:**
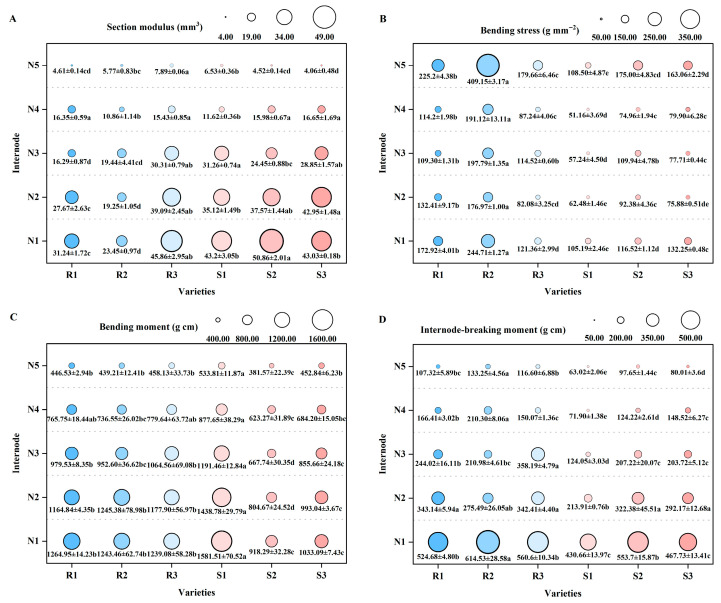
Comparison of main stem mechanical properties of different high-quality conventional rice varieties. (**A**) Section modulus (mm^3^); (**B**) bending stress (g mm^−2^); (**C**) bending moment (g cm); (**D**) internode-breaking moment (g cm). R1–R3 represent lodging-resistant varieties, S1–S3 represent lodging-susceptible varieties. N1–N5 represent the 1st to 5th internodes from the base upwards, respectively. Different lowercase letters represent statistically significant differences between varieties for the same indicator.

Bending stress, as an important indicator of material strength, reflects the quality of stem mechanics. The bending stress of each internode of the main stem showed a trend of greater stress in lodging-resistant varieties than in lodging-susceptible varieties, indicating that the mechanical properties of lodging-resistant varieties were better (Figure 4B). In addition, the section modulus of the N1-to-N3 internodes of the main stem of NX42 (R1) and LD3 (R2) is significantly lower than that of the lodging-susceptible varieties (Figure 4A). Meanwhile, between the N1-to-N4 internodes of the main stem, the bending moment of the lodging-resistant varieties was greater than that of HGNZ (S2) and HGYZ (S3). Among them, the main stem N2 internode bending moment of NX42 (R1), LD3 (R2), and SY17 (R3) was higher than that of HGNZ (S2) by 44.76%, 54.77%, and 46.38%, and higher than that of HGYZ (S3) by 17.30%, 25.41%, and 18.62% (Figure 4C). The internode-breaking moment of each variety decreases sequentially with the increase in internode position from N1 to N5. At the same time, the internode-breaking moment of N1, N4, and N5 internodes in LD3 (R2) were significantly higher than in JXSM (S1), reaching 42.69%, 192.49%, and 111.44%, respectively. The internode-breaking moment of N2 internodes in NX42 (R1) was significantly higher than that in JXSM (S1), reaching 60.41%. The internode-breaking moment of the N3 internode of SY17 (R3) was significantly higher than in JXSM (S1) by 188.75% (Figure 4D).

#### 2.3.2. Lodging Resistance

**Figure 5:**
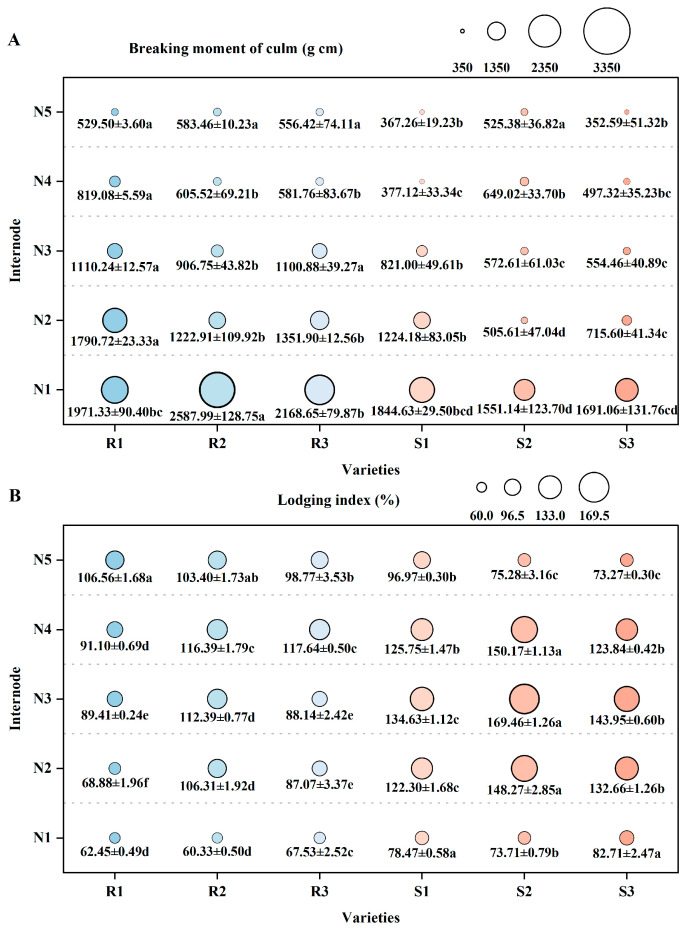
Comparison of lodging resistance of different high-quality conventional rice varieties. (**A**) Plant-breaking moment (g cm); (**B**) lodging index (%). R1–R3 represent lodging-resistant varieties, S1–S3 represent lodging-susceptible varieties. N1–N5 represent the 1st to 5th internodes from the base upwards, respectively. Different lowercase letters represent statistically significant differences between varieties for the same indicator.

The plant-breaking moment of each variety decreases sequentially with the increase in internode position from N1 to N5, too. Among different varieties, the plant-breaking moment at each internode of the main stem is greater in lodging-resistant varieties than in lodging-susceptible varieties. Meanwhile, in the internodes N1 to N4 of the main stem, the lodging index was significantly lower in lodging-resistant varieties than in lodging-susceptible varieties. Among them, the lodging index of N2 internodes in lodging-resistant varieties is 13.07–53.54% lower than that in lodging-susceptible varieties (Figure 5).

### 2.4. Comparison of Main Stem Yield-Related Traits and Panicle Characteristics of Different High-Quality Conventional Rice

#### 2.4.1. Main Stem Yield-Related Traits

**Figure 6:**
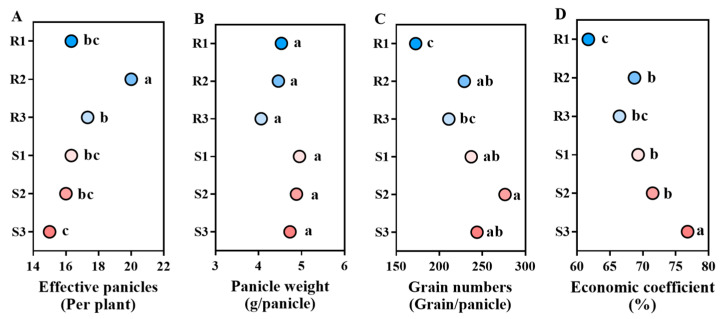
Comparison of yield-related traits of different high-quality conventional rice varieties. (**A**) Effective panicles (Per plant); (**B**) panicle weight (g/panicle); (**C**) Grain numbers (Grain/panicle); (**D**) Economic coefficient (%). R1–R3 represent lodging-resistant varieties, S1–S3 represent lodging-susceptible varieties. Different lowercase letters represent statistically significant differences between varieties for the same indicator.

The lodging-resistant varieties showed a trend of higher effective panicles than lodging-susceptible varieties, while panicle weight and grain numbers per panicle, and economic coefficient were lower than lodging-susceptible varieties. Among them, the effective panicles of LD3 (R2) were significantly higher than those of lodging-susceptible varieties by 22.47–33.33%. And the effective panicles of SY17 (R3) is 6.12–15.33% larger than that of the lodging-susceptible variety. The effective panicles of NX42 (R1) are greater than those of HGNZ (S2) and HGYZ (S3), but equal to those of JXSM (S1). The panicle weight and grain numbers per panicle, and economic coefficient of lodging-resistant varieties are lower than those of lodging-susceptible varieties by 4.22–17.98%, 3.38–29.22%, and 0.79–19.64%, respectively (Figure 6).

#### 2.4.2. Main Stem Primary Branch Traits

**Figure 7:**
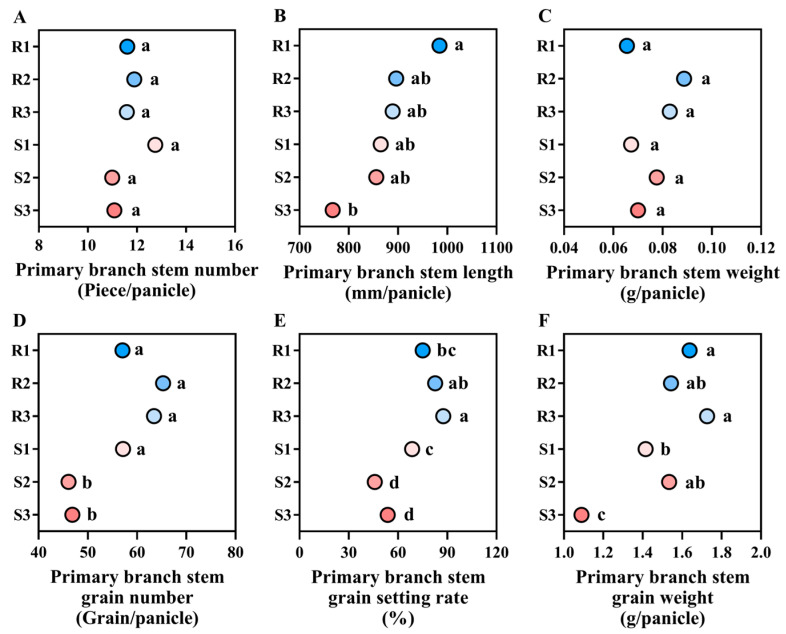
Comparison of main stem primary branch traits of different high-quality conventional rice varieties. (**A**) Primary branch stem number (Piece/panicle); (**B**) primary branch stem length (mm/panicle); (**C**) primary branch stem weight (g/panicle); (**D**) primary branch stem grain number (Grain/panicle); (**E**) primary branch stem grain setting rate (%); (**F**) primary branch stem grain weight (g/panicle). R1–R3 represent lodging-resistant varieties, S1–S3 represent lodging-susceptible varieties. Different lowercase letters represent statistically significant differences between varieties for the same indicator.

The differences in primary branch traits between lodging-resistant varieties and lodging-susceptible varieties are mainly manifested in primary branch stem length, grain setting rate, and grain weight. Among them, the primary branch stem length of the lodging-resistant varieties is 2.81–28.26% higher than the lodging-susceptible varieties. The primary branch stem grain setting rate of lodging-resistant varieties is 9.40–91.02% higher than the lodging-susceptible varieties. The primary branch stem grain weight of lodging-resistant variety is 0.61–58.60% higher than the lodging-susceptible variety. There was no significant difference in the primary branch stem number and weight between lodging-resistant varieties and lodging-susceptible varieties (Figure 7).

#### 2.4.3. Main Stem Secondary Branch Traits

**Figure 8:**
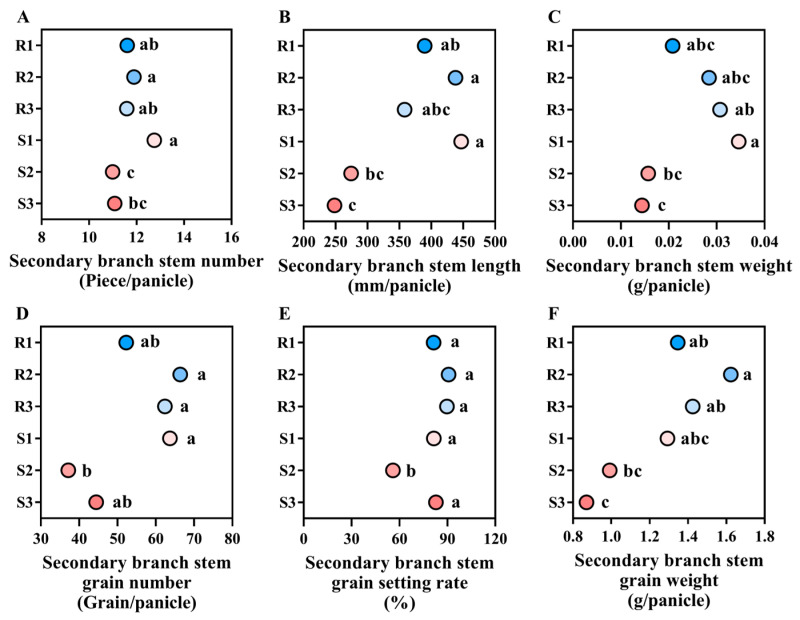
Comparison of secondary branch traits of different high-quality conventional rice varieties. (**A**) Secondary branch stem number (Piece/panicle); (**B**) secondary branch stem length (mm/panicle); (**C**) secondary branch stem weight (g/panicle); (**D**) secondary branch stem grain number (Grain/panicle); (**E**) secondary branch stem grain setting rate (%); (**F**) secondary branch stem grain weight (g/panicle). R1–R3 represent lodging-resistant varieties, S1–S3 represent lodging-susceptible varieties. Different lowercase letters represent statistically significant differences between varieties for the same indicator.

The differences in secondary branch traits between lodging-resistant varieties and lodging-susceptible varieties are mainly manifested in the secondary branch stem grain weight. The secondary branch stem grain setting rate of lodging-resistant varieties is 4.15–86.54% higher than lodging-susceptible varieties. There was no significant difference in the secondary branch stem number, length, weight, grain number, and grain setting rate between the lodging-resistant varieties and the lodging-susceptible varieties (Figure 8).

### 2.5. Correlation Analysis

**Figure 9:**
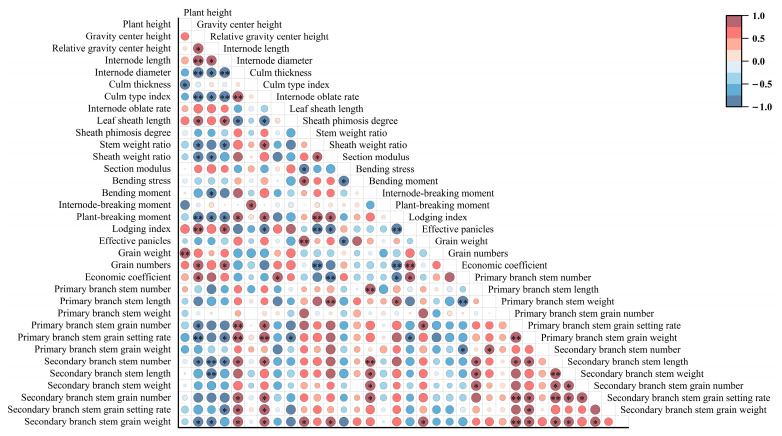
Correlation analysis was conducted on the main stem internode physical traits, stem and sheath plumpness traits, main stem mechanical properties, yield-related traits, and panicle characteristics of the N2 internodes. Note: * indicates significant difference (*p* < 0.05); ** indicates highly significant difference (*p* < 0.01).

Correlation analysis was conducted on the main stem internode physical traits, stem and sheath plumpness traits, main stem mechanical properties, yield-related traits, and panicle characteristics of the N2 internodes (Figure 9). We focused on the correlation between panicle characteristic indicators and stem lodging indicators. Based on the differences in internode length and culm thickness, further compare the culm type index and internode oblate rate of each internode of the main stem of different varieties. There is a highly significant positive correlation between plant height and panicle weight (r = 0.96 **). The gravity center height is significantly positively correlated with the grain numbers and the economic coefficient (r = 0.88 *, 0.83 *). And there is a significant or highly significant negative correlation with the primary branch stem grain number and grain setting rate; and secondary stem number and grain weight. Meanwhile, the relative gravity center height is significantly or highly significantly negatively correlated with the secondary branch stem number, length, and grain weight. The internode length is significantly positively correlated with the grain numbers (r = 0.87 *), while it is significantly negatively correlated with the primary branch stem grain setting rate, secondary branch stem number, and secondary branch stem grain setting rate. The internode diameter is significantly or highly significantly positively correlated with the primary branch stem grain number and grain setting rate; and secondary branch stem number, grain number, grain setting rate, and grain weight. There is a highly significant positive correlation between sheath phimosis degree and number of effective panicles (r = 0.98 **). The plant-breaking moment is significantly positively correlated with the primary branch stem length (r = 0.82 *), while it is significantly or highly significantly negatively correlated with the grain numbers and the economic coefficient (r = −0.95 **, r = −0.88 *). The lodging index is significantly or highly significantly positively correlated with grain numbers and economic coefficient (r = 0.97 **, r = 0.85 *), and significantly negatively correlated with the primary branch stem grain setting rate (r = −0.81 *). In addition, there is a significant or highly significant positive correlation between primary stem traits, secondary stem traits, and between primary and secondary stem traits.

### 2.6. Principal Component Analysis

**Figure 10:**
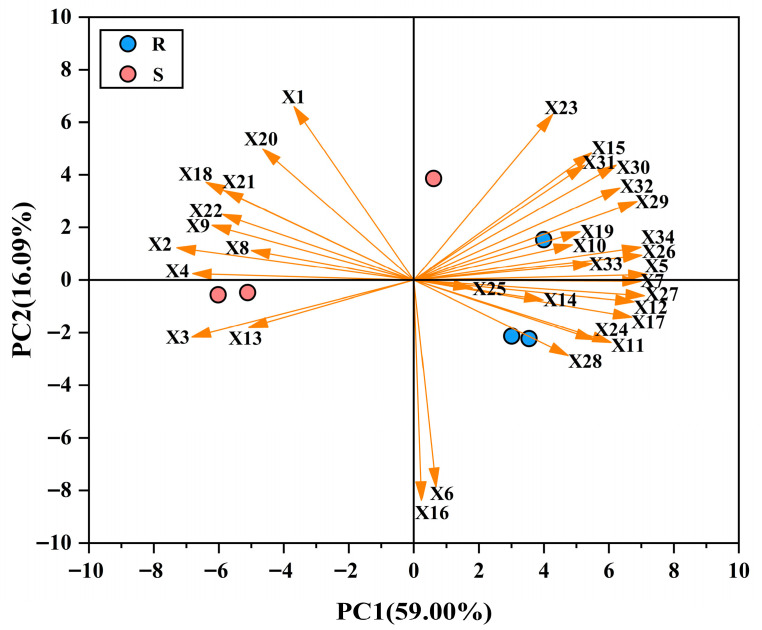
Principal component analysis was conducted on the main stem internode physical traits, stem and sheath plumpness traits, main stem mechanical properties, yield-related traits, and panicle characteristics of the N2 internodes. Note: X1: Plant height (cm); X2: Gravity center height (cm); X3: Relative gravity center height (%); X4: Internode length (cm); X5: Internode diameter (mm); X6: Culm thickness (mm); X7: Culm type index (%); X8: Internode oblate rate (%); X9: Leaf sheath length (cm); X10: Sheath phimosis degree (%); X11: Stem weight ratio (mg cm^−1^); X12: Sheath weight ratio (mg cm^−1^); X13: Section modulus (mm^3^); X14: Bending stress (g mm^−2^); X15: Bending moment (g cm); X16: Internode-breaking moment (g cm); X17: Plant-breaking moment (g cm); X18: Lodging index (%); X19: Effective panicles (per plant); X20: panicle weight (g/panicle); X21: Grain numbers (Grain/panicle); X22: Economic coefficient (%); X23: Primary branch stem number (Piece/panicle); X24: Primary branch stem length (mm/panicle); X25: Primary branch stem weight (g/panicle); X26: Primary branch stem grain number (Grain/panicle); X27: Primary branch stem grain setting rate (%); X28: Primary branch stem grain weight (g/panicle). X29: Secondary branch stem number (Piece/panicle); X30: Secondary branch stem length (mm/panicle); X31: Secondary branch stem weight (g/panicle); X32: Secondary branch stem grain number (Grain/panicle); X33: Secondary branch stem grain setting rate (%); X34: Secondary branch stem grain weight (g/panicle).

Principal component analysis of high-quality conventional rice varieties based on the lodging characteristics and panicle characteristics mentioned above. PCA1 and PCA2 account for 59.00% and 16.09% of the total variation, respectively. The cumulative contribution rate of the two is 75.09%. The PCA1 eigenvalue is 20.06, and the PCA2 eigenvalue is 5.47. Through PCA analysis, it was found that lodging-resistant varieties and lodging-susceptible varieties were clearly distinguished in PC1. The indicators of primary and secondary stem traits, and main stem mechanical properties, mainly cluster in the positive direction of PC1. And lodging-resistant varieties are gathered in this area. The main stem internode physical traits and yield-related traits are mainly in the negative direction of PC1. And the lodging-susceptible varieties gather in this area. Observing the distribution of indicators and combining the results of indicator significance analysis, it was found that plant height, relative gravity center height, internode diameter, leaf sheath phimosis, sheath weight ratio, bending stress, lodging index in stem characteristics; and effective panicles, grain numbers, primary branch stem length, primary branch stem grain setting rate, and secondary branch stem grain weight in panicle characteristics can all be important indicators for evaluating lodging performance of high-quality conventional rice (Figure 10).

### 2.7. Membership Function Analysis and Cluster Analysis

Based on the lodging resistance characteristic indicators and yield and panicle characteristic indicators mentioned above, the membership function values of each variety were calculated. Varieties based on the size of membership function values were ranked comprehensively. The higher the membership function value, the better the comprehensive trait performance of the variety. The membership function value of LD3 (R2) is the highest, at 0.77. This indicates that this variety performs well in rice lodging resistance and panicle yield. Next is SY17 (R3), with a membership function value of 0.68. The membership function value of HGNZ (S2) is the smallest, at 0.2. This indicates that the variety performs the worst in rice lodging resistance and panicle yield.

**Figure 11:**
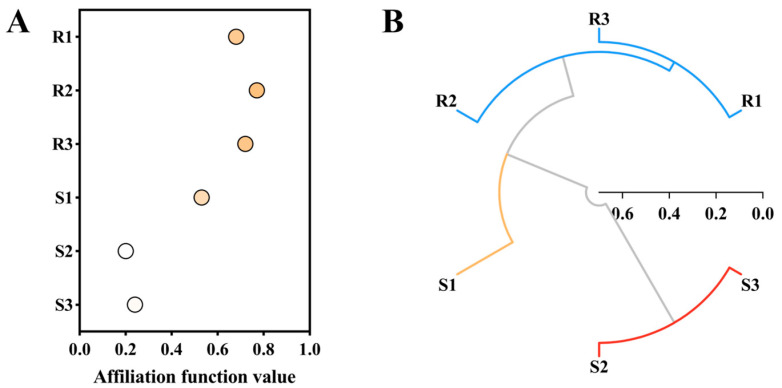
Membership function values and clustering results of high-quality conventional rice varieties. (**A**) Membership function analysis; (**B**) cluster analysis.

Further clustering analysis was conducted on six high-quality conventional rice varieties based on membership function values, and it was found that NX42 (R1), LD3 (R2), and SY17 (R3) were grouped together, and all showed lodging resistance in the field. The membership function values are all greater than or equal to 0.60. However, HGNZ (S2) and HGYZ (S3) are grouped together and are both varieties that exhibit lodging susceptibility in the field. The membership function values are all less than 0.30. In addition, the membership function value of JXSM (S1) is 0.53, which is separately clustered into one category. In the field, the lodging situation of this variety is intermediate, and the clustering results are consistent with reality. (Figure 11).

## 3. Discussion

Nowadays, consumers’ demand for nutrition, health, and food safety is becoming increasingly important. The cultivation of high-quality conventional rice is an indispensable choice to meet this demand. For breeding workers, this is also one of their important goals. However, the current complex and ever-changing climate has led to poor stability in factors such as temperature, humidity, and rainfall, posing severe challenges to the growth and development environment of rice. Rice lodging is one of the common phenomena that occur under extreme climate conditions. Once it occurs, it will not only make rice susceptible to pests and diseases but also affect nutrient absorption and photosynthesis during normal growth and development, seriously affecting the yield and quality of rice. The occurrence of lodging during the mature stage of rice will increase the difficulty of rice harvesting and increase yield losses. In case of rainy days or high humidity in the field, it is also easy to induce sprouting, decay, and deterioration on the panicle [30]. The occurrence of lodging during the heading or filling stage will directly affect the formation of rice grains. Taking CY-6 and Liangyoupeijiu as examples, for every day that lodging occurs earlier, it will cause a yield loss of 2.71% and 2.66%, respectively. The grain setting rate decreased by 1.8% and 2.1%, and the thousand grain weight decreased by 0.32 g and 0.27 g. At the same time, the rice quality was also affected, and the taste quality decreased [31]. If the rice is in the early stages of growth, the rice stalks are not soft enough and suffer extreme weather collapse, and the rice cannot even be harvested [32]. The same situation also occurs in wheat. The lodging during the heading and filling stages of wheat mainly causes an increase in infertile panicles, and a decrease in the grain number per panicle and in the thousand grain weight of winter wheat, which in turn leads to a decrease in yields. The lodging in the middle and late stages of grain filling mainly leads to a decrease in the thousand grain weight of winter wheat, while there is no significant change in the grain number per panicle [33]. This study focused on six high-quality conventional rice varieties and measured the related indicators of stem characteristics and panicle characteristics of each variety in order to comprehensively evaluate the factors affecting lodging of high-quality conventional rice and accurately assess the lodging resistance of varieties.

The physical properties of rice stems directly affect the ability of rice to resist wind and lodging. The results of this study indicate that lodging-resistant varieties have relatively lower plant height, gravity center height, and relative gravity center height. Specifically, the plant height of lodging-resistant varieties is 4.11–16.10% lower than that of lodging varieties, the gravity center height is 0.83–9.61% lower than that of lodging varieties, and the relative gravity center height is 0.09–12.68% lower than that of lodging varieties. We further compared the physical characteristics of each internode. The results showed that the difference in internode length was mainly concentrated in the second internode at the base (N2). Specifically, the internode length of lodging-resistant varieties N2 is less than that of lodging-susceptible varieties by 8.96–44.69%. However, there was no significant difference in internode length and panicle length. The relevant research results also indicate that the N2 internodes at the base of rice are the main internodes for bearing weight, especially in high-plant-height rice [34]. The cumulative results of N1-N2 and N3-N5 internodes still show that the length of N1-N2 internodes at the base of lodging-resistant varieties is smaller than that of lodging-susceptible varieties. This once again indicates that when improving the lodging resistance of rice varieties, the focus can be on the basal internodes. Reasonable internode length configuration can reduce the risk of rice lodging. In addition to genetic differences, factors affecting internode length changes include light intensity, leaf area, nitrogen application rate, and even the concentration of carbon dioxide in the environment [20,35,36]. In addition, the culm thickness and culm type index of the N1-to-N4 internodes at the base of the main stem were higher in lodging-resistant varieties than in lodging-susceptible varieties. But there was no significant difference in internode diameter. Tsugawa et al. found that there are differences in two traits among different cultivated varieties. One group of cultivated varieties has thicker but softer stems (thickness type), while another group of cultivated varieties has harder but thinner stems (hardness type). They refer to this change as the thickness–hardness trade-off. And a mechanical model was constructed to predict the lodging resistance of stems [37]. Therefore, the lodging resistant varieties in this study may be the thickness type mentioned in the aforementioned research.

The leaf sheath of rice is an important component of rice growth and development. But its effect on rice lodging resistance is often overlooked. The leaf sheath can tightly wrap around the stem, providing support and protection. This helps with the normal growth of stems. At the same time, leaf sheath also have a certain mechanical strength, which can increase the wind resistance of rice plants [38]. In this study, the length of the N1-N2 internodes of lodging-resistant varieties was smaller than that of lodging-susceptible varieties, and the length of the N1-N2 leaf sheath of lodging-resistant varieties was also smaller than that of lodging-susceptible varieties. But the sheath phimosis degree is greater than that of the lodging-susceptible varieties. We guess that leaf sheaths provide some support for lodging-resistant varieties. Joseph Cornwall et al. also found in wheat that increasing the radial preload force of the stem and the frictional force between the leaf sheath and the stem will increase the bending strength and stiffness of the wheat stem. This further enhances the lodging resistance of wheat [39]. Due to differences in stem and sheath length, culm thickness, and culm type index, there are also differences in the stem weight ratio and sheath weight ratio of N2 internodes at the base between lodging-resistant and lodging-susceptible varieties. Specifically, the N2 internodes at the base of lodging-resistant varieties are 16.37–268.58% heavier than the stem weight ratio of lodging-susceptible varieties, and 8.27–165.01% heavier than the sheath weight ratio of lodging-susceptible varieties. The research results of Zhang et al. also showed that although rice has a smaller stem thickness and thinner wall thickness, the dry weight of the leaf sheath per centimeter is larger, and the proportion of cellulose and lignin in the stem is higher, which enhances the stem strength of rice [40].

Although there is no clear pattern in the section modulus between lodging-resistant and lodging-susceptible varieties, the bending stress and internode-breaking moment of each internode in the main stem show a trend of lodging-resistant varieties being greater than lodging-susceptible varieties. Researchers have also enhanced the mechanical strength of rice stems by improving their resistance to bending and breaking moments through reasonable nitrogen fertilizer treatment [41]. Similar research is also reflected in the study of maize lodging resistance. Spraying ethylene can change the morphological characteristics of corn internodes and improve the stem strength of corn [42]. Even results have shown that the structure of corn stems is more predictive of stem bending strength than their chemical composition [43]. The plant-breaking moment is also a comprehensive indicator of the ability of rice to withstand their own external loads. Our results still show that the plant-breaking moment in N1–N3 of lodging-resistant varieties are greater than those of lodging-susceptible varieties. The lodging index reflects the strength of a plant’s ability to resist lodging. The smaller the lodging index, the stronger its ability to resist lodging [24]. The lodging index in N1–N4 internodes of lodging-resistant varieties is lower than that of lodging-susceptible varieties. Therefore, we speculate that the stem mechanical properties of lodging-resistant varieties are better, with better strength and toughness to cope with lodging.

This study also compared the main stem yield and panicle traits of high-quality conventional rice varieties. The results showed that the effective panicles of lodging-resistant rice varieties were greater than those of lodging-susceptible varieties, while the panicle weight, grain numbers, and economic coefficient were lower than those of lodging-susceptible varieties. Varieties that experience lodging have higher panicle weight, grain numbers, and economic coefficient. Further analysis was conducted on the primary and secondary branch traits of the panicle. It is interesting that lodging-resistant varieties have higher primary and secondary stem grain setting rate and primary and secondary stem grain weight. Therefore, we speculate that lodging-resistant varieties have a more reasonable panicle structure. Although varieties with lodging have greater yield potential, improper panicle structure may make them more prone to lodging, directly affecting grain filling and maturation, and thus affecting yield formation. The study by E Santosa et al. also showed that if rice lodging occurs in the late stage of grain filling, the yield decreases by 10–20%. In the early stage of grouting, due to an increase in empty particles and a lower thousand particle weight, the yield will decrease by 50–60%, or even more severely [44]. This brings up another contradiction. The improvement of rice yield requires an increase in plant height and an increase in biological yield to achieve the goal of increasing rice economic yield. But an increase in plant height and yield will increase the risk of lodging. Once lodging occurs, there is a possibility of reduced production. Some cultivators have proposed some cultivation measures to address this contradiction. The relevant measures include the application of silicon potassium fertilizer, and the use of calcium hexanedione and uniconazole, which can increase yield and reduce lodging risk [45]. By promoting preheading growth of rice to regulate biomass production and transport, lodging resistance can be improved while maintaining high yield [46]. Maintain high rice yield and improve lodging resistance of rice plants through wet dry alternating irrigation method [47]. Based on our results, we guess whether it is possible to reduce the risk of lodging while stabilizing overall yield by increasing effective panicles and reducing the panicle weight and grain numbers per panicle.

The correlation analysis results show that there is a significant or highly significant correlation between stem characteristic indicators and panicle characteristic indicators, including gravity center height, relative gravity center height, internode length, internode diameter, stem weight ratio, sheath weight ratio, bending moment, plant-breaking moment, and lodging index, etc. At the same time, principal component analysis based on these indicators distinguishes between lodging-resistant varieties and lodging-susceptible varieties. The research results of Li et al. showed that upright and semi upright panicle varieties have shorter plant height and shorter internodes at the base, which is more conducive to lodging resistance. Its panicles are short, light, and have a small curvature at the panicle neck, resulting in minimal force on the stem [15]. But it is not necessarily the case that curved panicle types have poorer lodging resistance than upright ear types. The panicle shape affects lodging resistance through bending moment and gravity center height. When the bending moment increases and the gravity center height rises, the resistance to lodging weakens, and vice versa, the resistance to lodging strengthens. Indica rice, especially large panicle hybrid indica rice, has a short panicle neck and drooping panicle. The curvature of the panicle neck is greater than 120°, the gravity center height is reduced, and the bending moment is reduced, which is beneficial for improving lodging resistance [48]. So, the lodging resistance trait of rice is a complex characteristic involving stem and panicle characteristics. In research, close attention should be paid to the connection between the two. The membership function evaluation method is often used for comprehensive performance evaluation of optimal processing [49,50,51]. And cluster analysis is achieved by processing similar backgrounds within clustering analysis [36,52,53]. This study comprehensively evaluated and classified six high-quality conventional rice varieties through membership function method and cluster analysis. The results showed that the membership functions of NX42 (R1), LD3 (R2), and SY17 (R3) were all greater than 0.60, and they were grouped together as low-risk lodging varieties. The membership function value of JXSM (S1) is 0.53, which is classified as a medium-risk variety for lodging. The membership functions of HGNZ (S2) and HGYZ (S3) are both greater than 0.30, and they are grouped together as high-risk varieties for lodging.

This study explores the relationship between lodging and material heritability in rice. But related studies also indicate that agronomic measures play an important role in improving rice lodging resistance. By regulating water management (such as alternating wet and dry irrigation), fertilization management (such as nitrogen fertilizer application), and cultivation techniques (such as reasonable planting and timely sun drying), the lodging resistance of rice can be significantly improved [54,55,56]. For example, compared to the drought flood alternation stress treatment during the tillering stage, the drought flood alternation stress treatment during the jointing stage can reduce the photosynthetic capacity of post-flowering leaves and rice panicle weight. This will improve the plumpness of the stem and increase the safety factor of stem lodging resistance [47]. In addition, the application of growth regulators such as paclobutrazol has been shown to enhance the lodging resistance of rice [57]. Therefore, future research should further explore the interaction between agronomic measures and genetic factors to achieve comprehensive improvement of rice lodging resistance. This not only helps to improve the yield and quality of rice but also provides theoretical support for rice lodging resistance breeding and cultivation management.

## 4. Materials and Methods

### 4.1. Experimental Material

The experimental materials consisted of the following 6 high-quality conventional rice varieties: “Nongxiang 42” (NX42; R1), “Longdao 3” (LD3; R2), “Songya 17” (SY17; R3), “Jianxiangsimiao” (JXSM; S1), “Huangguangnongzhan” (HGNZ; S2), “Huangguangyinzhan” (HGYZ; S3). The selected varieties are all high-quality conventional rice varieties widely planted in the middle and lower reaches of the Yangtze River. All varieties are excellent cultivated species approved by the national or provincial authorities. All seeds were provided by the Rice Research Institute of Hunan Agricultural University. The varieties showed non-lodging in R1–R3 and lodging in S1-S1 at 30 days after full heading. Each variety’s main stem contains the same six specific parts: five internodes (numbered N1 to N5 from base to top) and one panicle (*p*).

### 4.2. Experimental Design

**Figure 12:**
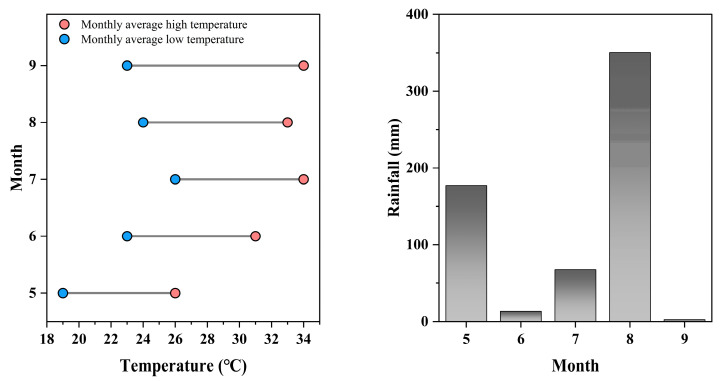
Monthly average temperature and average rainfall in the relevant experimental areas. (**A**) Monthly average high temperature and low temperature; (**B**) onthly average rainfall.

This study was conducted in Longping Rice Planting Park, Lukou Town, Changsha County, Hunan Province in 2021 (113°23′ E, 28°42′ N). Basic physical and chemical properties of soil in the cultivated layer (0–20 cm) of the experimental site: organic matter is 31.2 g/kg, total nitrogen is 0.3 g/kg, total phosphorus is 0.6 g/kg, available phosphorus is 10.2 mg/kg, available potassium is 216.5 mg/kg, available nitrogen is 385.5 mg/kg, pH is 5.3. The monthly average temperature and average rainfall in the relevant experimental area are shown in Figure 12. The plot area of each variety was set at 80 m^2^. Each variety has three replicates, and each experimental unit is arranged in random block. Adopt manual transplanting method to transplant the seedlings at 25 days of age. Transplant density is 25 cm × 14 cm. The fertilizer program was nitrogen fertilizer (150 kg·hm^−2^) applied in stages as base fertilizer: tiller fertilizer: panicle fertilizer = 4:3:3. Phosphorus fertilizer (75 kg·hm^−2^) was applied in full as basal fertilizer. Potassium fertilizer (75 kg·hm^−2^) is applied with 50% base fertilizer and 50% panicle fertilizer. Field management measures are implemented according to local high-yield cultivation requirements. At the heading stage, mark 30 main stems with consistent panicles by hanging tags. At 30 days after heading, 20 main stems with uniform panicle were collected from the labeled plants in each plot. Investigate the main stem internode physical traits, stem and sheath plumpness traits, main stem mechanical properties, and lodging resistance-related indicators. In addition, the number of effective panicles in 6 varieties with consistent growth was investigated during the rice maturity period. And indicators related to main stem yield-related traits and panicle characteristics were examined.

### 4.3. Determination Indicators and Methods

#### 4.3.1. Main Stem Internode Physical Traits

Determination of plant height, gravity center height and calculation of relative gravity center height according to the methods of Wang et al. [47], Lai et al. [14], Xiao et al. [58] is illustrated with the following formula:

Relative gravity center height (%) = (Gravity center height (cm)/Plant height (cm)) × 100(1)

Fresh main stem internodes were transected, measuring the external and internal diameters of the long and short axes of the internodes. Use a_1_ and a_2_ to represent the external and internal diameters of the minor axis of the internode (mm), respectively. Use b_1_ and b_2_ to represent the external and internal diameters of the major axis of the internode (mm), respectively. To calculate internode diameter, clum thickness, clum type index, and internode oblate rate, the formula is

Internode diameter (mm) = (a_1_ + b_1_)/2 (2)

Clum thickness (mm) = [(a_1_ + b_1_) − (a_2_ + b_2_)]/4 (3)

Clum type index (%) = [Internode diameter (average of major and  minor axes, cm)/Culm length (cm)] × 100(4)

Internode oblate rate (%) = [1 − (Minor axis of internode (mm)/ Major axis of internode (mm))] × 100(5)

#### 4.3.2. Stem and Sheath Plumpness Traits

Measure the internode length, the leaf sheath length, and the length of each leaf sheath from the point of attachment to the opening of the leaf sheath. Weigh single stem internodes, leaves, and leaf sheaths for fresh weight. To calculate sheath phimosis degree, ratio of culm weight, and ratio of sheath weight, the formula is

Sheath phimosis degree (%) = Length from leaf sheath attachment  to leaf sheath opening (cm)/Leaf sheath length (cm) × 100(6)

Stem weight ratio (mg cm^−1^) = [Fresh weight of internode (g)/ Internode length (cm)] × 1000(7)

Sheath weight ratio (mg cm^−1^) = [Fresh weight of leaf sheath (g)/ Leaf sheath length (cm)] × 1000(8)

#### 4.3.3. Main Stem Mechanical Properties

Internode bending resistance and plant bending resistance were determined using a stem strength tester (YYD-1, Top Instruments Co., Ltd., Hangzhou, China) according to the method of Lei et al. [59] and Ookawa et al. [60]. Calculate the indexes related to the mechanical properties of stems.

Section modulus (mm^3^) = (a_1_^3^ b_1_ − a_2_^3^ b_2_)/4a_1_ (9)

Bending stress (g mm^−2^) =10 × Internode-breaking moment (g cm)/ Section modulus (mm^3^) (10)

Bending moment (g cm) = Fresh weight from the base  of the tested internode to the panicle top (g) × Length  from the base of the internode to the panicle top (cm)(11)

Lodging index (%) = Bending moment (g cm)/ Plant-breaking moment (g cm) × 100.(12)

#### 4.3.4. Yield-Related Traits and Panicle Characteristics

Determine the panicle length of the main stem, determine the panicle weight, panicle numbers, and filled panicle numbers per panicle, and calculate the seed setting rate and thousand grain weight. Investigate the number, length, weight, filled panicle numbers, panicle weight, and seed setting rate of primary and secondary branches in the panicle.

### 4.4. Statistical Analysis

Microsoft Excel 2021 was used for data organization. DPS data processing system was used for analysis of significance of differences and Ducan’s new complex polarity method for significance testing. SPSS 25.0 was used for correlation analysis, principal component analysis, membership function analysis, and cluster analysis (hierarchical clustering). The Graph Pad Prism 9 and Origin 2023 were used for graphing.

## 5. Conclusions

Lodging-resistant high-quality conventional rice varieties had a more reasonable internode structure, lower plant height, gravity center height, and relative gravity center height, shorter and thicker N2 internode morphology, higher sheath phimosis degree, harder bending stress, internode-breaking moment, plant-breaking moment, and smaller lodging index compared to lodging-susceptible high-quality conventional rice varieties. However, research on lodging resistance characteristics still needs to focus on rice panicle traits. In this study, the effective panicles of lodging-resistant varieties were greater than those of lodging-susceptible varieties, while the panicle weight, grain numbers, and economic coefficient were lower than those of lodging-susceptible varieties. We guess whether it is possible to reduce the risk of lodging while stabilizing overall yield by increasing effective panicles and reducing the panicle weight and grain numbers per panicle. In addition, the lodging performance of the varieties obtained through membership function evaluation and cluster analysis in this study is consistent with the actual field performance. The membership function evaluation and cluster analysis can be used as analytical methods for evaluating the lodging resistance of high-quality conventional rice.

## Data Availability

The original contributions presented in the study are included in the article; further inquiries are available from the corresponding author on reasonable request.
